# Radioembolization Planning With Dual-Isotope Acquisition of ^166^Ho-Labeled Microparticles and ^99m^Tc-Mebrofenin

**DOI:** 10.1097/RLU.0000000000004732

**Published:** 2023-06-08

**Authors:** Sándor Czibor, András Bibok, Dénes Horváthy, Krisztián Fábián, Tamás Györke

**Affiliations:** From the Departments of ∗ Nuclear Medicine; † Interventional Radiology; ‡ Radiology, Medical Imaging Centre, Semmelweis University; § Mediso Medical Imaging Systems Ltd, Budapest, Hungary.

**Keywords:** radioembolization, dual-isotope, holmium, mebrofenin, functional liver volumetry

## Abstract

A 76-year-old man with hepatocellular carcinoma was referred for liver radioembolization. Given a prior left hemihepatectomy, it was clinically important to consider potentially irradiated healthy liver at planning. Thus, at the SPECT/CT imaging of the scout dose ^166^Ho-microparticles before injected superselectively in the right hepatic artery, ^99m^Tc-mebrofenin was injected intravenously, and functional volumetry SPECT was performed simultaneously. Based on the 2 image sets, the nonirradiated healthy liver was calculated as 1589 mL (functional liver reserve of 85.5% on ^99m^Tc-mebrofenin SPECT). Posttreatment dosimetry calculations showed optimal normal tissue and tumor absorbed doses, and the patient is clinically well after 3 months.

**FIGURE 1:**
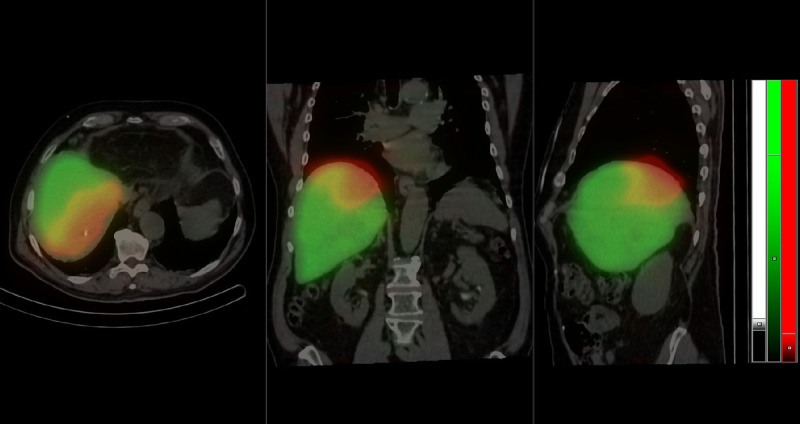
Fused ^99m^Tc-mebrofenin (green) and ^166^Ho (red) dual-isotope SPECT and CT images, transversal left panel, coronal middle panel, and sagittal right panel. Images were acquired on a Mediso SCP SPECT/PET/CT triple-modality camera (Mediso Medical Imaging Systems Ltd) equipped with a triple-detector SPECT after the transarterial injection of 140 MBq ^166^Ho-microspheres (QuiremScout; Quirem Medical B.V.) in the angiographic laboratory and the subsequent IV administration of 270 MBq of ^99m^Tc-mebrofenin. The healthy liver tissue included in the potential irradiation target of the planned radioembolization is marked by the yellow penumbra on the SPECT/CT images. The majority of the liver, however, expresses only ^99m^Tc-mebrofenin uptake (green), and the target encompassing the tumor is covered by the deposition of ^166^Ho-microspheres (red). Volumes of interests were drawn around the respective isotope distributions, and calculations from the encompassed volumes showed an estimated functional liver remnant (%FRL-C) of 85.5%, which was critically important considering that the patient had previously undergone a left hemihepatectomy. Fifteen days after the scout imaging, the radioembolization treatment was performed with 5 GBq of ^166^Ho-microspheres (QuiremSpheres; Quirem Medical B.V.) injected transarterially from the same catheter position. Posttreatment ^166^Ho-SPECT/CT images showed an identical microsphere deposition to that of the scout image set, and dosimetric evaluation showed an estimated mean absorbed dose of 37 Gy in the healthy liver and of 199 Gy in the tumor. Hepatic radioembolization with ^166^Ho-labeled polylactate acid spheres allows the pretreatment scout performed with identical spheres as to the treatment particles and the images to be acquired by SPECT, using the gamma emission of ^166^Ho (6.6% abundance of 81 keV photons).^1,2^ The intrahepatic distribution of ^99m^Tc-mebrofenin identifies healthy liver tissue, and it has been used for volumetric measurements before liver surgery to calculate total and future remnant liver volume^3–7^ and it is increasingly used in the workup of radioembolization procedures.^8,9^ The feasibility of dual-isotope acquisition of ^99m^Tc-colloid and ^166^Ho-microspheres has been shown^10,11^; however, we suggest that hepatocyte-specific mebrofenin is more suitable for future liver remnant investigations in the dual-isotope imaging setting than labeled colloids that are proportional to the activity of the reticuloendothelial system.^12,13^ Here, we presented a case underlining that dual-isotope acquisition of ^99m^Tc-mebrofenin and ^166^Ho is clinically feasible and the complementary information gained from this examination helps to plan radioembolization strategies where future remnant liver function is equivocal. This dual-isotope SPECT/CT acquisition has the advantage of imaging the patient in the same position and that only one low-dose CT scan is necessary for attenuation-correction compared with the setting where ^99m^Tc-mebrofenin functional volumetry and ^166^Ho-scout SPECT/CT is performed at different time points.

**FIGURE 2:**
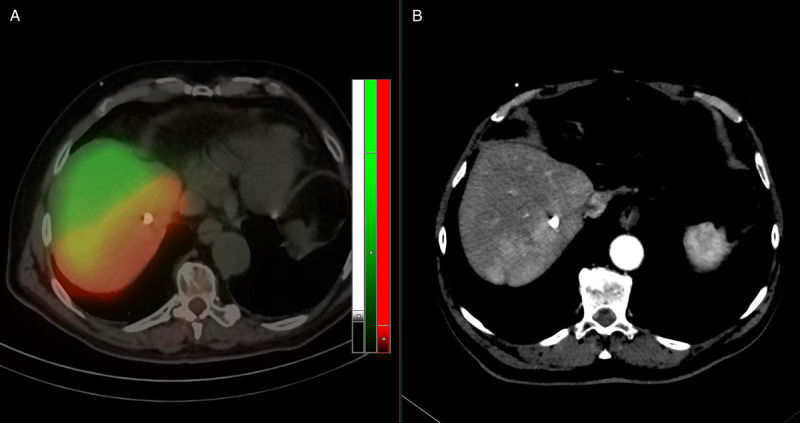
Fused ^99m^Tc-mebrofenin (green) and ^166^Ho (red) dual-isotope SPECT and CT images (**A**) with the corresponding transaxial slice of the arterial phase of the diagnostic CT scan acquired 12 days before (**B**), showing the contrast-enhancing tumor.

**FIGURE 3:**
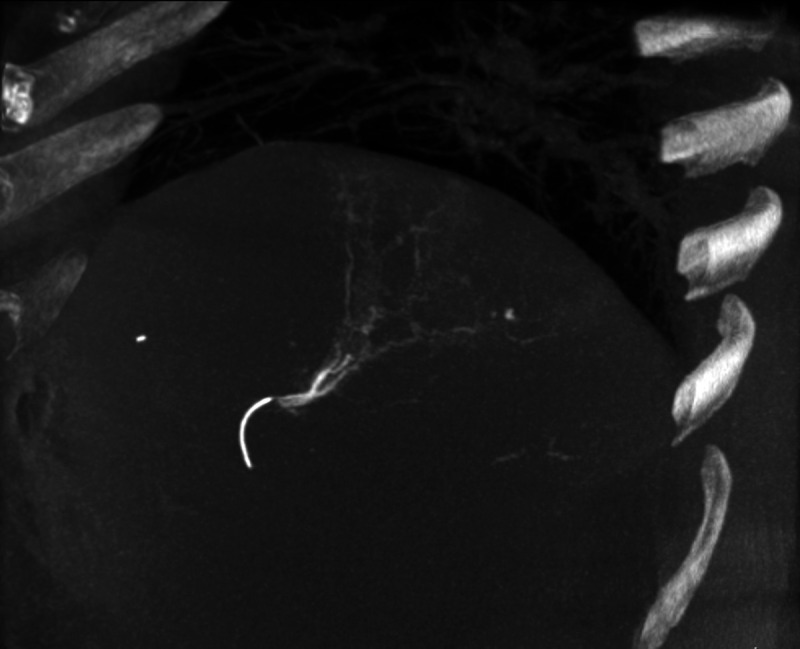
Selective cone-beam CT angiogram (sagittal plane, 50 mm MIP reconstruction) of the ^166^Ho-microspheres injection position using a microcatheter in the S VII segmental branch of the hepatic artery. The patient’s renal function remained normal on laboratory tests 1 week before and after the angiography (blood urea nitrogen: 12.6 mg/dL vs 15.7 mg/dL; creatinine: 0.72 mg/dL vs 0.74 mg/dL).
